# Evidence of low within‐pair genetic relatedness in a relict population of Thorn‐tailed Rayadito despite long‐term isolation

**DOI:** 10.1002/ece3.8679

**Published:** 2022-03-07

**Authors:** Esteban Botero‐Delgadillo, Verónica Quirici, Silvina Ippi, Rodrigo A. Vásquez, Bart Kempenaers

**Affiliations:** ^1^ Department of Behavioural Ecology and Evolutionary Genetics Max Plank Institute for Ornithology Seewiesen Germany; ^2^ Departamento de Ciencias Ecológicas Facultad de Ciencias Instituto de Ecología y Biodiversidad Universidad de Chile Santiago Chile; ^3^ SELVA: Research for Conservation in the Neotropics Bogotá Colombia; ^4^ Departamento de Ecología y Biodiversidad Facultad de Ciencias de la Vida Universidad Andrés Bello Santiago Chile; ^5^ Centro de investigación para la sustentabilidad Universidad Andrés Bello Santiago Chile; ^6^ Departamento de Zoología CRUB Universidad Nacional del Comahue – CONICET Bariloche Argentina

**Keywords:** Chile, inbreeding avoidance, kinship, natal dispersal, random mating

## Abstract

Investigating whether mating patterns are biased in relation to kinship in isolated populations can provide a better understanding of the occurrence of inbreeding avoidance mechanisms in wild populations. Here, we report on the genetic relatedness (*r*) among breeding pairs in a relict population of Thorn‐tailed Rayadito (*Aphrastura spinicauda*) in north‐central Chile that has experienced a long‐term history of isolation. We used simulations based on 8 years of data to assess whether mating is random with respect to relatedness. We found that mean and median population values of pair relatedness tended to be lower than randomly generated values, suggesting that mating is not random with respect to kinship. We hypothesize that female‐biased dispersal is the main mechanism reducing the likelihood of mating among kin, and that the proportion of related pairs (i.e., *r* > 0.125) in the study population (25%) would presumably be higher in the absence of sex‐biased dispersal. The occurrence of other mechanisms such as extra‐pair copulations, delayed breeding, and active inbreeding avoidance through kin discrimination cannot be dismissed and require further study.

## Graphical abstract

We studied an isolated, relict population of Thorn‐tailed Rayadito, a socially monogamous songbird that exhibits female‐biased dispersal. Using breeding and genetic data collected during 2010–2017, we found that mean and median population values of within‐pair genetic relatedness tended to be lower than randomly generated values, which indicates that mating is not random with respect to kinship in our study population. Although we did not directly assess the relationship between dispersal and pair relatedness, we hypothesize that female dispersal could act as a passive mechanism to reduce the probability of close inbreeding.

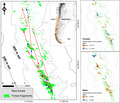

## 1 INTRODUCTION

Inbreeding can negatively affect populations by increasing homozygosity and thereby the expression of deleterious recessive alleles, potentially leading to a reduction in fitness that is commonly referred to as inbreeding depression (Charlesworth & Charlesworth, 1999; Keller & Waller, 2002). These effects may select for various mechanisms of inbreeding avoidance in wild populations (Pusey & Wolf, 1996), although the prevalence and strength of such mechanisms are highly variable across species and are largely contingent on historical, demographic, and ecological factors (de Boer et al., 2021; Duthie & Reid, 2016; Pike et al., 2021). Inbreeding avoidance mechanisms include active mate choice mediated by kin‐recognition, delayed maturation or reproductive suppression, extra‐pair or extra‐group copulations, and sex‐biased dispersal (Pusey & Wolf, 1996).

Recent literature reviews and meta‐analyses suggest that inbreeding avoidance might not be prevalent in wild populations and that unbiased mating with regard to kinship appears widespread across several animal taxa (de Boer et al., 2021; Pike et al., 2021). However, as inbreeding depression seems more frequent in smaller or isolated populations (Crnokrak & Roff, 1999), mechanisms to avoid incestuous mating are more likely to be found under such circumstances (see e.g., Pike et al., 2021). Besides the conservation value of studying inbreeding in small and isolated populations (Frankham, 1998), investigating whether mating patterns are biased in relation to kinship in populations with known historical, demographic, and ecological contexts can be insightful. For instance, it can help to better understand the circumstances that favor the occurrence of inbreeding avoidance in the wild.

**FIGURE 1:**
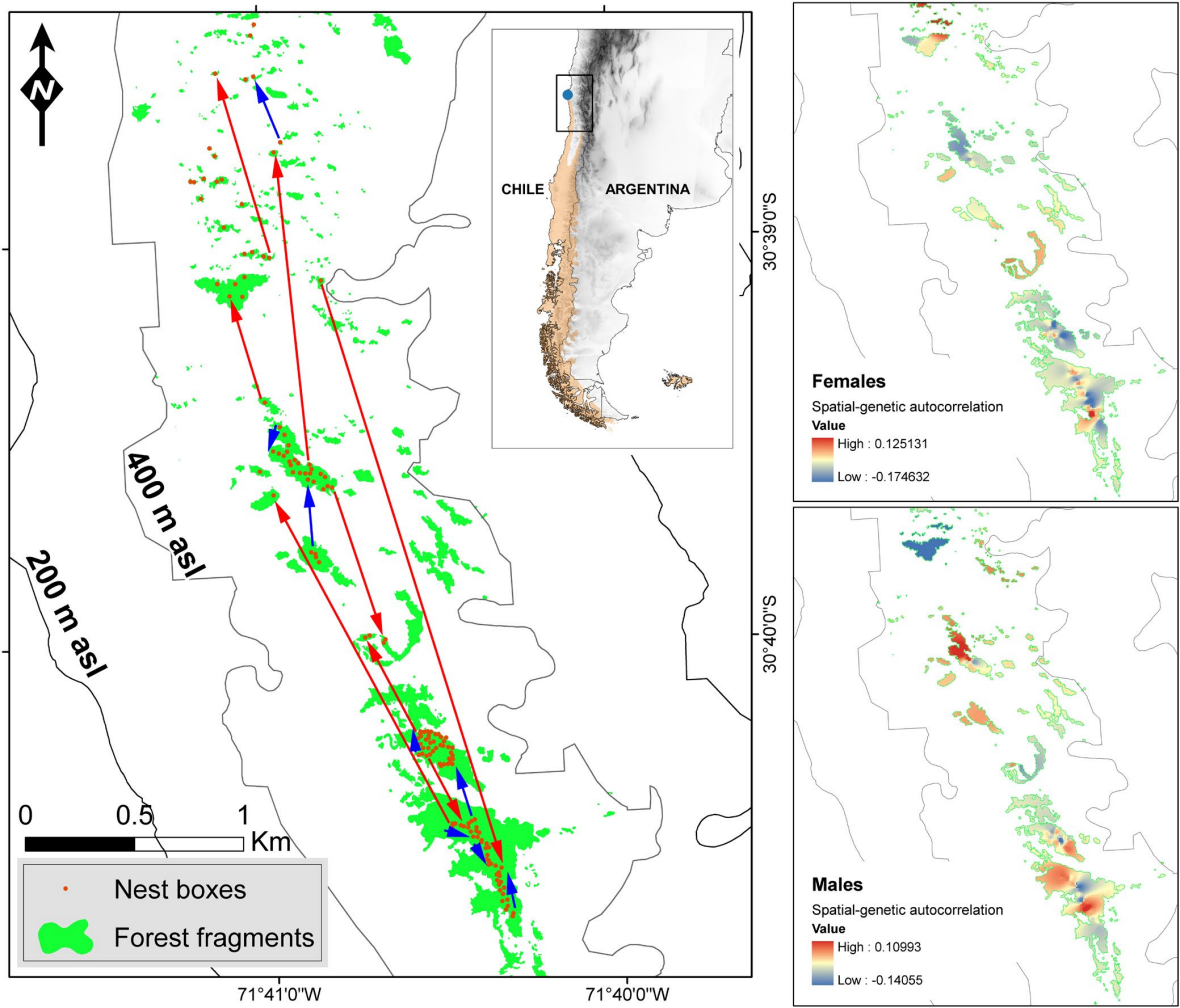
Sex‐specific local dispersal patterns and resulting fine‐scale genetic landscapes in an isolated population of Thorn‐tailed Rayadito in Fray Jorge National Park, north‐central Chile. The panel on the left illustrates the distribution of forest fragments and nestboxes used to monitor the breeding biology of the species. Arrows illustrate the female‐biased natal dispersal pattern observed in the population: red arrows exemplify the movements typically observed in post‐fledging females, and blue arrows represent movements described for post‐fledging males (documented median distances: females = 780 m; males = 85 m; see more details in Botero‐Delgadillo et al., 2017, 2019). The two panels on the right depict sex‐specific genetic landscapes that result from the local dispersal pattern, showing values of spatial‐genetic autocorrelation for each sex for 2010–2015. Genetic autocorrelation maps were produced by extrapolating values generated via 2D spatial autocorrelation analyses as described in Botero‐Delgadillo et al. (2017). Input data for autocorrelation analyses were deposited by Botero‐Delgadillo et al. (2018)

Here, we combine 8 years of breeding monitoring and genetic data to assess whether mating is random with respect to genetic relatedness in an isolated population of a socially monogamous bird, the Thorn‐tailed Rayadito (*Aphrastura spinicauda*). The study population inhabits a temperate forest relict in Bosque Fray Jorge National Park in north‐central Chile, breeding in tree cavities in forest fragments that are surrounded by a semi‐arid landscape (del‐Val et al., 2006; Figure 1). Although post‐glacial aridisation have caused the study population to experience a long‐term process of isolation since the end of the tertiary (Villagrán et al., 2004), it harbors a relatively high level of genetic diversity (observed heterozygosity, *H*_O_ =0.67) and a moderate level of inbreeding (population mean inbreeding coefficient, *F* = 0.18), and it has been suggested that, along with historical factors, female‐biased natal dispersal could account for such level of inbreeding (Botero‐Delgadillo et al., 2019; Botero‐Delgadillo, Quirici, Poblete, Acevedo, et al., 2020; Botero‐Delgadillo, Quirici, Vásquez, et al., 2020).

Female rayaditos in Fray Jorge disperse from their natal site and end up breeding in nearby or distant forest fragments, whereas males often establish their breeding territories close to their natal area and rarely leave the fragment where they fledged (Botero‐Delgadillo et al., 2019). This creates a characteristic genetic landscape where the spatial distribution of females is not associated with their degree of genetic relatedness, while males show fine‐scale genetic autocorrelation (Botero‐Delgadillo et al., 2017; Figure 1). Whether this translates into an unbiased mating pattern with respect to relatedness in this population remains unknown. If female‐biased natal dispersal is reducing the risk of mating with close kin, we would expect lower relatedness among breeding pairs than expected under random mating (van Tienderen & van Noordwijk, 1988; but see Pärt, 1996 for a discussion on the use of random mating as a null model). We here compare observed values of relatedness among breeding pairs with expected values generated under random mating, and discuss whether results are consistent with the observation of female‐biased dispersal.

## 2 MATERIALS AND METHODS

### 2.1 Study population

Data were collected in Bosque Fray Jorge National Park (30°38′S, 71°40′W), north‐central Chile (Figure 1), as part of a long‐term study on the breeding biology of rayaditos. A total of 101–157 nest boxes were installed in forest fragments since 2007, which are monitored each year between September and December to record reproductive phenology and productivity. The semiarid landscape in Fray Jorge is dominated by xerophytic vegetation, and rayaditos inhabit relicts of Valdivian temperate forest that persist atop the coastal mountain range as humid conditions are maintained year‐round due to oceanic fog‐water inputs (del‐Val et al., 2006). This population is located at the northern limit of the species distributional range, and gene flow between this and other continental populations is limited (Botero‐Delgadillo, Quirici, Poblete, Acevedo, et al., 2020).

Rayaditos in Fray Jorge lay one clutch of 1–4 eggs per season during the austral spring (Moreno et al., 2005), although replacement clutches after complete nest failure can occur. Birds start breeding between 1 and 3 years of age, and yearly apparent survival for adults is estimated at 68% (Botero‐Delgadillo et al., 2017). Some individuals have been recorded up to nine years after the first capture. Reproductive pairs establish territories during the breeding season, and nest‐site fidelity (~70%; see Botero‐Delgadillo et al., 2017) and mate fidelity (~58%; E. Botero‐Delgadillo, unpublished) are relatively high in this population. Natal dispersal is restricted by forest fragmentation, and it presumably takes place during the non‐breeding season, when breeding territories are dissolved (Vergara & Marquet, 2007) and rayaditos form conspecific flocks (Ippi & Trejo, 2013) that wander across the study area.

### 2.2 General procedures

We captured and marked all nestbox occupants (adults and nestlings) with numbered aluminum rings when nestlings were 12–14 days old. We used mist nets to capture adults breeding in natural cavities within the study site. Blood samples (~15 µl) were obtained from all individuals by puncturing the brachial vein with a sterile needle and subsequently stored on FTA™ Classic Cards (Whatman™) for genetic analyses. We extracted DNA from blood samples for genotyping and molecular sexing following the protocol described in Botero‐Delgadillo et al. (2017). We genotyped a total of 183 breeding adults at 12 autosomal, polymorphic microsatellite loci (for details see Botero‐Delgadillo et al., 2017; Botero‐Delgadillo, Quirici, Vásquez, et al., 2020). We determined sex by amplifying the CHD locus using the primers P2/P8 (Griffiths et al., 1998). Microsatellite amplifications were performed in multiplex PCRs using the Type‐it^®^ Microsatellite PCR Kit (QIAGEN^®^ #206246) and primer mixes containing four to five primer pairs (mix 1, 2, and 3; see Botero‐Delgadillo et al., 2017). Alleles were assigned using the GeneMapper 4.0 software (Applied Biosystems). In order to minimize genotyping error, ambiguous samples were genotyped repeatedly until achieving consistent peak readings, and allele assignment was performed independently by two different observers.

### 2.3 Genetic analysis and estimation of relatedness

We first tested for deviations from Hardy–Weinberg equilibrium (HWE) and linkage disequilibrium (LD) in our population using the packages adegenet (Jombart, 2008) and poppr (Kamvar et al., 2014) in the free software R 4.0.2 (R Core Team, 2020). There were no deviations from HWE after applying Bonferroni correction for multiple comparisons (all *p* > .1), nor evidence for LD between any pair of loci (standardized index of association, ${\bar{r}}_{d}$ = 0.0042). The frequency of null alleles in the sampled population was <0.03 for all loci, thus no samples or loci were excluded. The probability of identity across loci with our marker set was 9.65 × 10^−12^.

**FIGURE A1:**
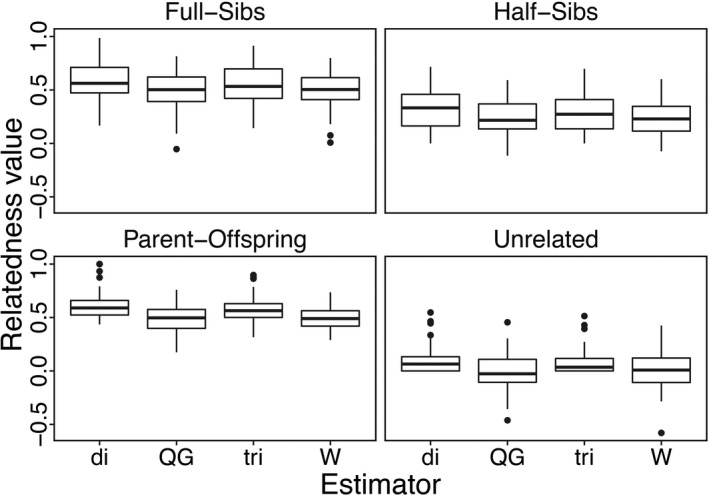
Correlation between expected and observed genetic relatedness values (*r*) for 100 generated pairs of individuals for each degree of relatedness (parent‐offspring PO, full sibs FS, half‐sibs HS, and unrelated UR) based on observed allelic frequencies in a population of Thorn‐tailed Rayadito. Four estimators are compared: the dyadic likelihood estimator (di), the Queller and Goodnight estimator (QG), the triadic likelihood estimator (tri), and the Wang estimator (W)

We used the R package related (Pew et al., 2015) to estimate pairwise genetic relatedness (hereafter *r*). Genotyping error rates were fixed to 0, and given that our marker panel consisted of less than 15 loci, we did not account for close inbreeding (see Wang, 2011). We assumed that close inbreeding was rare or absent because the frequency of mating among close relatives in the study population is rather low (see *Results*). Initially, we calculated four estimators of *r*, namely the Q&G estimator (Queller & Goodnight, 1989), the Wang estimator (Wang, 2002), the dyadic likelihood estimator (Milligan, 2003), and the triadic likelihood estimator (Wang, 2007). We conducted simulations using the *familyism* function to assess estimator performance. We generated 100 pairs of individuals for each degree of relatedness (parent‐offspring PO, full sibs FS, half‐sibs HS, and unrelated UR) based on observed allelic frequencies, and then estimated the correlation between observed and expected values. All estimators performed similarly, but the triadic likelihood estimator (hereafter trioML) showed a slightly higher correlation coefficient (Figure A1), and hence was selected as a measure of *r*. Finally, we used the *coancestry* function to calculate pairwise *r*‐values between all genotyped birds using 500 reference individuals (i.e., an individual used as a control to estimate *r* between two other individuals) as suggested by Wang (2007).

### 2.4 Statistical analyses

We calculated the genetic relatedness for all breeding pairs captured between 2010 and 2017 (*n* = 179). Given that some pairs were repeatedly sampled during the course of the study, we included only the first observation of each pair to avoid biasing the estimated population mean/median estimates of the pair *r* values. Thus, subsequent analyses were performed on a set of 101 unique breeding pairs.

To assess whether mating is random with respect to relatedness, we calculated the probability of obtaining the observed population mean and median pair *r* values under a scenario of random mating. For this, we randomly assigned a male partner to every sampled female in our population and subsequently calculated the mean and median pair *r* value. We generated 10,000 random sets of 101 breeding pairs to produce a distribution of simulated *r* values and to estimate the two‐sided *p*‐value for the observed mean and median values. We used sampling without replacement for our simulations, constraining pairs to be formed only between individuals that were captured during the same year but across all forest fragments, independent of location. This way, we ensured that pairs would be formed between birds that (i) were reproductively active during the same breeding season and (ii) that were not already mated. We repeated the analyses described above for each individual year, this time reducing the number of random sets to 1,000 and the sample sizes according to the number of pairs that were actually captured during each year (see Table 1). Additionally, breeding pairs that remated during any given year were forced to remain paired. Simulations of pair formation were performed using a custom routine in R that is made available along with the analyzed data (see Data Availability).

**TABLE 1:** Yearly values of the population mean and median genetic relatedness (*r*) of breeding pairs were observed during 2010–2017 in an isolated population of Thorn‐tailed Rayadito

Year	*n*	Obs. mean *r*	Probability	Obs. median *r*	Probability
2010	12	0.094 (0.095)	.48	0.082 (0.074)	.65
2011	22	0.073 (0.099)	.04	0.055 (0.062)	.31
2012	22	0.083 (0.094)	.23	0.048 (0.062)	.19
2013	25	0.108 (0.099)	.73	0.059 (0.065)	.36
2014	18	0.113 (0.095)	.12	0.033 (0.076)	.02
2015	26	0.082 (0.103)	.09	0.033 (0.070)	<.0001
2016	22	0.096 (0.099)	.43	0.017 (0.079)	<.0001
2017	32	0.084 (0.108)	.07	0.041 (0.081)	<.005

Note

The number of breeding pairs genotyped (*n*) is specified for each year. Observed mean and median values were obtained from a distribution of 1000 simulated values, and the probability of obtaining such values under a scenario of random mating with respect to kinship is indicated. Expected mean and median values under random mating are in parentheses.

## 3 RESULTS

The observed population mean and median pair relatedness for the 2010–2017 period were *r* = 0.084 (SD = 0.12) and *r* = 0.036 (interquartile range, IQR = 0.11), respectively. Pair *r* values were >0.125 for 25 genotyped breeding pairs (25%), and >0.25 for 11 pairs (11%). We estimated *r* values ~.5 for two breeding pairs. One of these cases involved a male that mated with its mother 1 year after its father disappeared from the study population. This pair bred together for at least five consecutive years. The other breeding pair with a high *r*‐value could have been a PO or an FS pair, but we could not confirm this with our capture‐mark‐recapture data.

**FIGURE A2:**
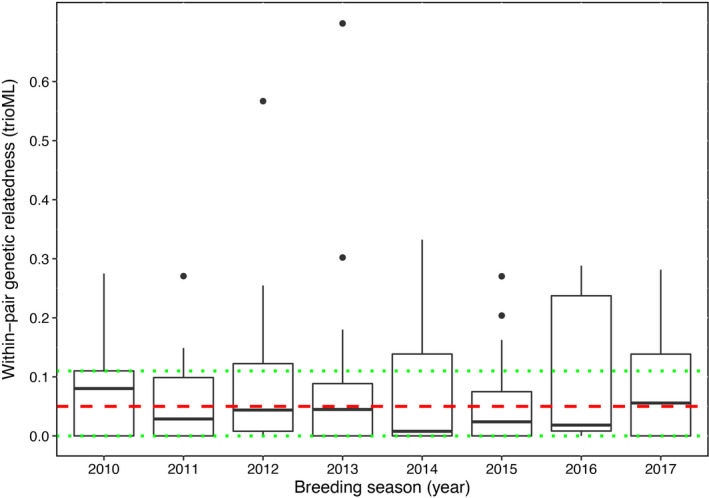
Boxplots showing yearly variation in genetic relatedness (*r*) among breeding pairs in a population of Thorn‐tailed Rayadito studied during 2010–2017. The triadic likelihood estimator was used to calculate *r*. The red dashed line represents the overall median value calculated for 101 unique breeding pairs that were captured during the study period. The green dotted lines show the overall interquartile range (IQR)

Pair *r* values did not vary substantially between years. With the exception of 2014 and 2016, when the population median *r* was closer to zero and the IQR was relatively large, the within‐year variance was similar during most of the study (Figure A2).

**FIGURE 2:**
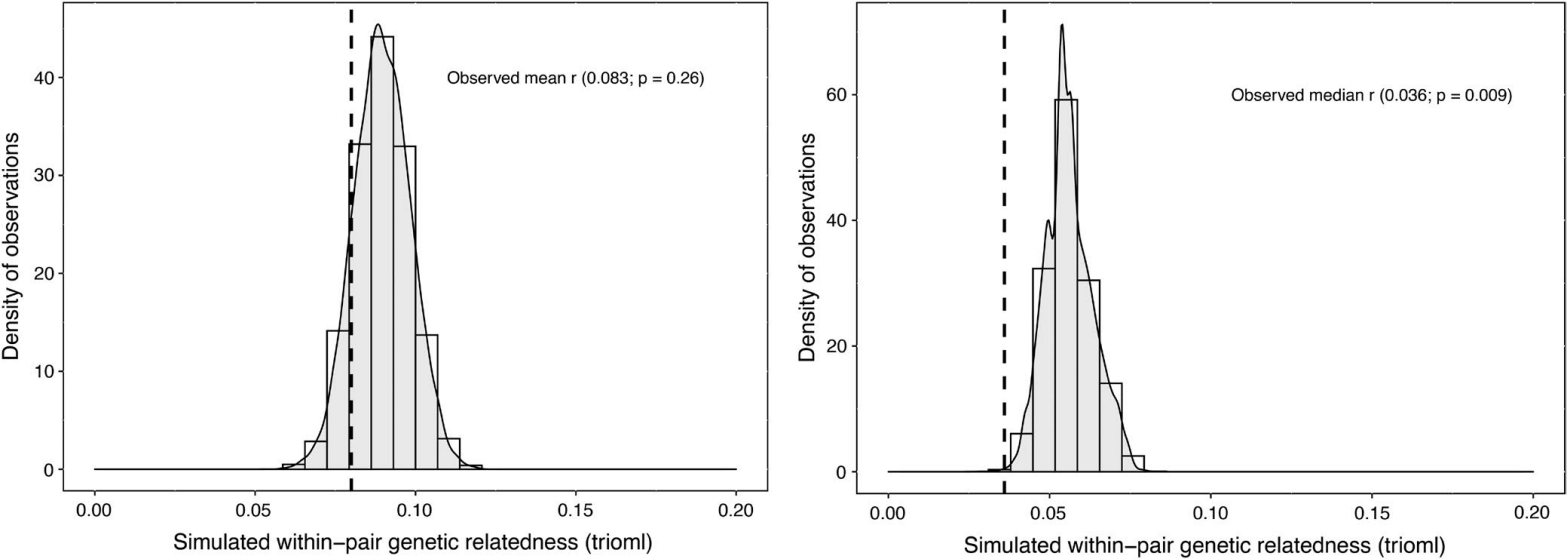
Distribution of pair genetic relatedness values (*r*) generated by simulating 10,000 random sets of breeding pairs of Thorn‐tailed Rayadito based on 101 pairs that were captured during 2010–2017 in Fray Jorge National Park, north‐central Chile. The Left and right panels show the distribution of simulated mean and median values, respectively. The actually observed values (dashed lines) and their two‐sided *p*‐value are included

Our analysis based on all captured breeding pairs showed that both the observed mean and median pair *r* values fell on the lower tail of the distribution of simulated *r* values (Figure 2). If mating would be random with respect to relatedness during the study period, the probability of observing the population mean and median values would be 0.26 and 0.009, respectively. Yearly analyses supported these results, with mean and median observed values falling on the left tails of the simulated distributions for almost all years (Table 1). The probability of obtaining the observed values under random mating ranged from 0.04 to 0.73 for the population mean, and from 0 to 0.65 for the median (Table 1).

## 4 DISCUSSION

Based on breeding data collected during 2010–2017 and genetic analyses, we found that mean and median values of the genetic relatedness (*r*) between breeding pairs tended to be lower than expected under random mating in an isolated population of Thorn‐tailed Rayadito. Non‐random mating with regard to kinship might partially account for why this relict population still exhibits a relatively high level of genetic diversity and a level of inbreeding that could be considered lower than expected given its long‐term history of isolation. The mechanisms that could be operating to reduce inbreeding in this population are briefly discussed below.

**FIGURE A3:**
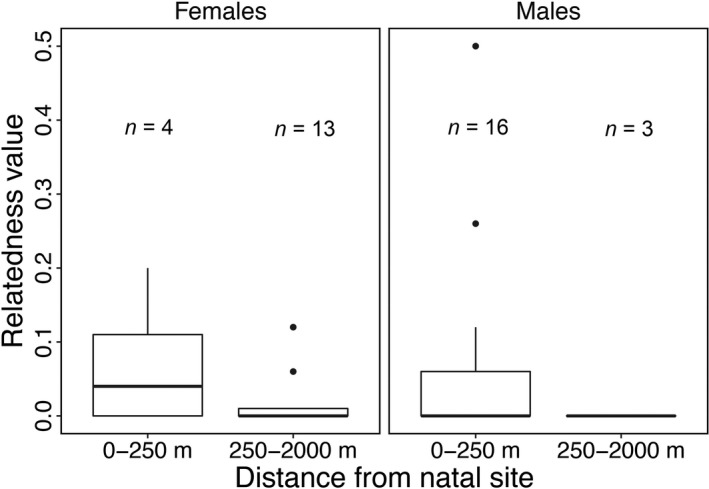
Variation in genetic relatedness (*r*) among breeding pairs for a subset of female and male Thorn‐tailed Rayaditos for which natal dispersal distance was estimated using capture‐mark‐recapture data collected during 2010–2017. As variation in *r* values was reduced, birds were grouped into two categories depending on whether they typically dispersed close to their natal site (i.e., no farther than five territories away) or within the same natal forest fragment (frequently from 0 to 250 m), or farther from their natal site or to a different forest fragment (frequently more than 250 m)

There is evidence indicating that the rayadito population in Fray Jorge National Park has undergone a long period of gradual isolation and demographic stability (Botero‐Delgadillo, Quirici, Poblete, Acevedo, et al., 2020). This alone could be sufficient to explain the observed levels of genetic diversity and inbreeding, but passive or active behavioral mechanisms might also keep inbreeding at bay. Previous studies have suggested that female‐biased dispersal in Fray Jorge could play an important role as a strategy to avoid inbreeding (Botero‐Delgadillo et al., 2019; Botero‐Delgadillo, Quirici, Vásquez, et al., 2020). We were unable to directly assess whether the dispersal history of breeding birds correlated with pair relatedness because we had information on natal dispersal distances for only ~10% of all captured individuals (see Botero‐Delgadillo et al., 2017, 2019). However, a close inspection of natal dispersal data for those individuals revealed that birds that dispersed farther from their natal site or to another forest fragment (typically >250 m) tended to show lower values of within‐pair *r* (Figure A3). Moreover, although related pairs (*r* > 0.125) comprised almost a quarter of the breeding population, this proportion would presumably be higher in the absence of sex‐biased dispersal.

We cannot dismiss the possibility that other mechanisms also reduce inbreeding in this population. First, extra‐pair copulations, which are higher in this than in other rayadito populations (e.g., 21% of broods contain extra‐pair offspring vs. 14% in a population in southern Chile; Botero‐Delgadillo, Quirici, Poblete, Ippi, et al., 2020), could help reduce the incidence of inbreeding (see Rowley et al., 1993). Delayed breeding could also be an important mechanism, as recapture data indicate that more than 50% of all individuals may start their first breeding attempt during their second or third year of life (Botero‐Delgadillo et al., 2017). Probably a consequence of competition for mates and/or breeding sites in this densely populated environment, some birds might be forced to postpone breeding while dispersing away from their closest kin in order to procure a mate or a nest site (Botero‐Delgadillo et al., 2017, 2019). Active inbreeding avoidance through kin discrimination could be operating in rayaditos as well and deserves further study, although evidence for such mechanism in wilds birds is rare (see Legibre et al., 2010 and references therein).

There are some caveats regarding our methodology that need to be acknowledged. Given the wide use of randomization tests to assess whether mating is random with respect to relatedness in wild bird populations (Eikenaar et al., 2008; Gibbs & Grant, 1989; Legibre et al., 2010; Pärt, 1996; van Tienderen & van Noordwijk, 1988), we here adopted random mating as the null model. We are, however, fully aware that some of its underlying assumptions may be too simplistic. For instance, our null model implied that both females and males dispersed randomly and were able to pair with any unmated individual of the opposite sex, assuming that (i) all individuals had an equal probability of dispersing and (ii) all birds paired more or less simultaneously during a given breeding season (see Gibbs & Grant, 1989; van Tienderen & van Noordwijk, 1988). Although incorporating dispersal probabilities into the null model would have been preferable, we lacked data on natal dispersal distances for the vast majority of sampled individuals (see above).

Despite the assumptions and limitations of our randomization approach, it is important to highlight that random mating is a rather conservative null hypothesis—and thus difficult to reject. For instance, in closed populations where the probability of mating with unrelated individuals is 10 times larger than mating with close kin, a random mating null model would lead to an expected frequency of close inbreeding of 1% or less (Pärt, 1996). The most likely conclusion under such a scenario will be that close inbreeding is not avoided, even when it actually is. In our population, 25% of all pairings were between related birds (*r* > 0.125), while 11% were between close kin (*r* > 0.25). Still, ~70% of all pairwise *r* values among all genotyped rayaditos were <.09 (Figure S1), and ~60% of all possible breeding pairs that could be randomly formed were between unrelated individuals (*r* < 0.05). This means that in our study population, the probability to mate with an unrelated bird is high even under random mating, and hence the power to reject the null hypothesis is likely low. Yet, we found evidence supporting the hypothesis that mating was not random for relatedness.

**FIGURE A4:**
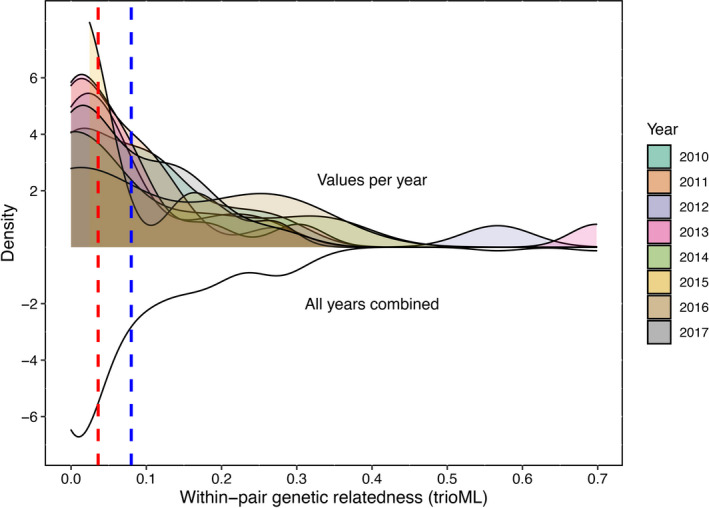
Density distribution of within‐pair genetic relatedness (*r*) in a population of Thorn‐tailed Rayaditos. Density distributions are shown for the entire study period (2010–2017) and for each year. Global mean (blue dotted line) and median (red dotted line) *r* values are shown

Statistical support for non‐random mating with regard to relatedness was more conclusive for the median than for the mean *r* coefficient. However, the fact that the observed values were consistently lower than simulated values with both metrics indicates that our findings might not be an artifact. Although the mean *r* value is the most commonly used metric in inbreeding studies (see e.g., Foerster et al., 2006; Gibbs & Grant, 1989; Leedale et al., 2020), median values can be a better measure of central tendency in some instances, particularly when variation around *r* is high and the mean is affected by “extreme” values. In our study population, for instance, most pairs consisted of unrelated individuals, but ~25% of all pairings occurred among related birds or close kin with moderate to high *r* values. As this likely shifted the population mean value farther away from the peak of the distribution, the median seemed a more representative metric of the within‐pair global and yearly *r* values (Figure A4).

Our results indicate that mating between genetically related rayaditos in Fray Jorge occurs less often than would be expected under random mating, and we hypothesize that female‐biased dispersal is the main mechanism reducing the likelihood of mating among kin. It is possible that a high historical genetic diversity and a stable demographic history allowed this population to maintain low levels of homozygosity, but the existence of active and/or passive strategies to avoid incestuous matings is also plausible. A recent study in Fray Jorge showed that female rayaditos with moderate levels of genetic diversity have slightly higher reproductive success than highly homozygous or heterozygous females, meaning that mechanisms to avoid inbreeding and outbreeding could have evolved in this population (Botero‐Delgadillo, Quirici, Vásquez, et al., 2020). It is likely that both historical (e.g., the presence of bottlenecks) and ecological (e.g., sex‐biased dispersal or extra‐pair copulations) factors are important modulators of inbreeding levels, and comparative studies focused on populations with different demographic histories may be valuable to determine their relative contribution and their prevalence in nature.

## CONFLICT OF INTEREST

None declared.

## AUTHOR CONTRIBUTIONS

**Esteban Botero‐Delgadillo:** Conceptualization (lead); Data curation (lead); Formal analysis (lead); Funding acquisition (supporting); Investigation (lead); Methodology (equal); Project administration (equal); Writing – original draft (lead). **Verónica Quirici:** Data curation (supporting); Funding acquisition (supporting); Investigation (supporting); Methodology (equal); Resources (supporting); Writing – review & editing (supporting). **Silvina Ippi:** Funding acquisition (supporting); Methodology (supporting); Resources (supporting); Writing – review & editing (supporting). **Rodrigo A. Vasquez:** Funding acquisition (lead); Investigation (supporting); Resources (lead); Supervision (supporting); Writing – review & editing (supporting). **Bart Kempenaers:** Conceptualization (supporting); Funding acquisition (supporting); Investigation (supporting); Project administration (equal); Resources (supporting); Supervision (lead); Writing – original draft (supporting); Writing – review & editing (lead).

### OPEN RESEARCH BADGES

The data and the code used for all analyses are deposited in the Dryad Digital Repository: https://doi.org/10.5061/dryad.qnk98sfjk.

## Supporting information

Figure S1

## Data Availability

All data analyzed in this study and code for replicating the analyses are available from the Dryad Digital Repository (https://doi.org/10.5061/dryad.qnk98sfjk).
