# Supplementary material for: Evidence of low within‐pair genetic relatedness in a relict population of Thorn‐tailed Rayadito despite long‐term isolation
Source: Ecol Evol. 2022 Mar 7;12(3):e8679. doi: 10.1002/ece3.8679 (PMC8901872; doi:10.1002/ece3.8679)

Pairwise genetic relatedness *r* (trioML) estimated for 183 genotyped individuals of Thorn-tailed Rayadito from Fray Jorge National Park, north-central Chile

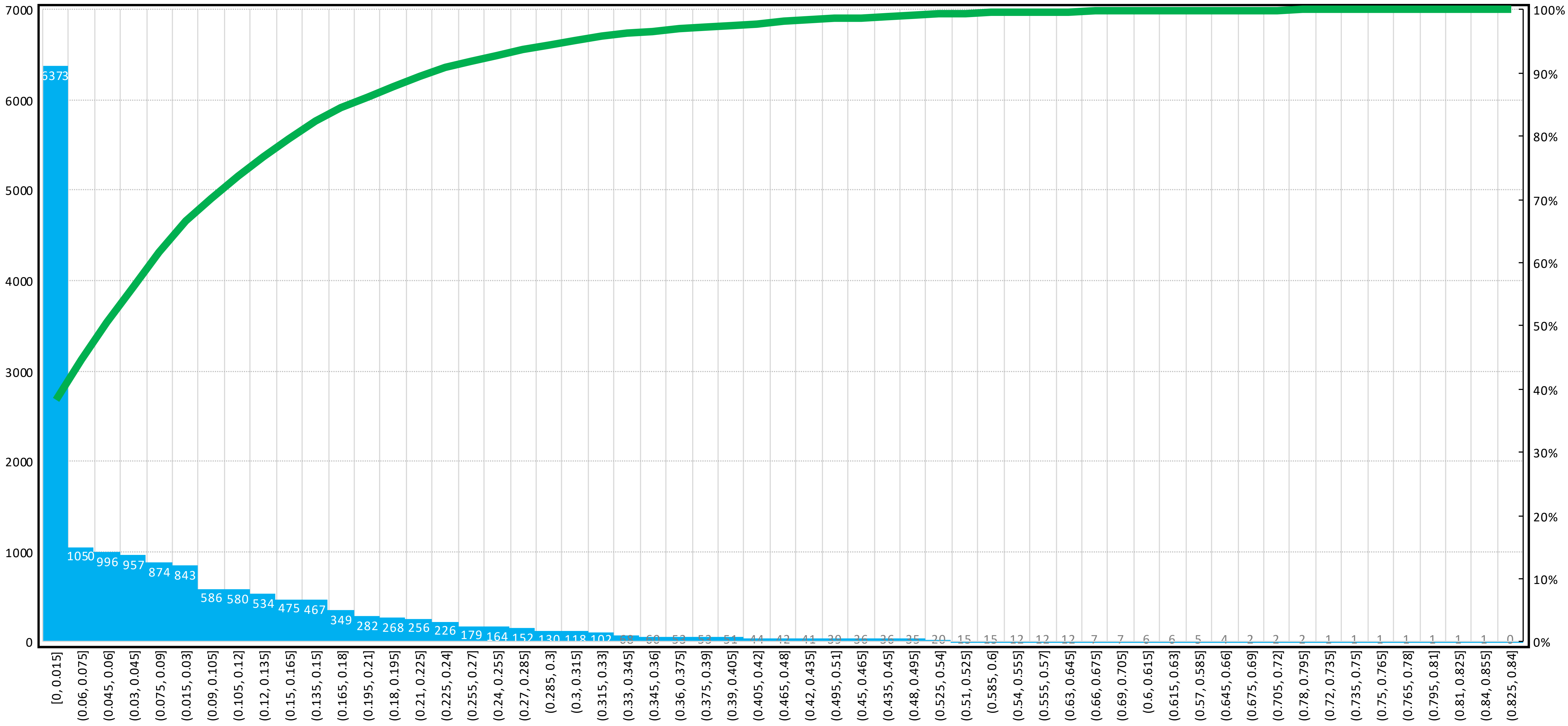

Supplement: Supplementary file 1 — Figure S1 [file ECE3-12-e8679-s001.pdf]
